# A Pioneer Scheme

**Published:** 1920-10-23

**Authors:** 


### A Pioneer Scheme.

### EAST LANCASHIRE TUBERCULOSIS SETTLEMENT.

East Lancashire has just adopted a scheme for a tuberculosis industrial colony and village settlement which is of great interest. The local seventy-five per cent. of funds contributed in response to the Red Cross appeal will be devoted by the East Lancashire Committee to this scheme, which has been submitted to the Ministry of Health for their approval.

The details which are given in the *Manchester Guardian* are that an estate of 150 acres of farm and park land has been purchased at Barrowmore, in one of the pleasantest and healthiest spots in Cheshire. It was bought for £19,000, and the Committee of the East Lancashire Branch are now asking for £100,000 to enable them to carry on there the work of training tuberculous ex-soldiers, putting out into employment those that prove themselves fit to work, and keeping in a village settlement on the estate those who are deemed unfit to take employment outside.

It is hoped that when Barrowmore is in working order 200 men may be accommodated. It is not proposed to take to the estate men who obviously are beyond hope of recovery: the men the settlement will deal with are those who are fit to work, but unfit to work in ordinary labour conditions and for the customary hours of labour. The treatment will not be sanatorium treatment; there will be none of the open-sided sheds and other such things associated with sanatoria. The settlement will lie "between sanatorium and home life." Strict regard will be had to living conditions in the light of the latest medical knowledge of tuberculosis; but at the same time rules and restrictions are to be reduced to a point where they will in no wise hamper a normal life. When, for example, the village is founded upon the estate there will be a shop and a chapel, and in the cottages the men will live with their wives and families the ordinary life of a country village.

There will be the training block of buildings, the hospital block, and the village settlement. The men in the first case will come into the training block. A committee is being formed to discuss the question of trades; but there are in mind such subjects as woodwork, french-polishing, basket making, brush making, leather work, repoussé brass and copper work, market gardening, poultry and pig keeping. When the men have spent twelve or eighteen months in training, those that are fit will be drafted out to work at home, under a system of after-care. Those who show that they will never be physically fit for competing in the ordinary labour market will be passed on to the village settlement, the married men living in cottages with their families, the single in hostels. They will be employed permanently in the workshops or gardens of the estate. It is hoped to begin with twenty cottages, and this number will be added to as funds permit.

It is hoped that building will begin next month, and that some patients will be in by the new year.

A scheme of this sort involving quite a new idea in the treatment of consumptive patients, must have the public sympathy and support if it is to succeed. Barrowmore will keep the men at work; it will help them to see how they may help themselves.

